# Evidence that minocycline treatment confounds the interpretation of neurofilament as a biomarker

**DOI:** 10.1093/braincomms/fcaf175

**Published:** 2025-05-23

**Authors:** Juliana E Gentile, Christina N Heiss, Taylor L Corridon, Meredith A Mortberg, Stefanie Fruhwürth, Kenia Guzman, Lana Grötschel, Laia Montoliu-Gaya, Kwan Chan, Neil C Herring, Timothy Janicki, Rajaa Nhass, Janani Manavala Sarathy, Brian Erickson, Ryan Kunz, Alison Erickson, Craig Braun, Katherine T Henry, Lynn Bry, Steven E Arnold, Eric Vallabh Minikel, Henrik Zetterberg, Sonia M Vallabh

**Affiliations:** Program in Brain Health, Broad Institute of MIT and Harvard, Cambridge, MA 02142, USA; Department of Psychiatry and Neurochemistry, Institute of Neuroscience and Physiology, Sahlgrenska Academy at the University of Gothenburg, Mölndal 431 80, Sweden; Program in Brain Health, Broad Institute of MIT and Harvard, Cambridge, MA 02142, USA; Program in Brain Health, Broad Institute of MIT and Harvard, Cambridge, MA 02142, USA; Department of Psychiatry and Neurochemistry, Institute of Neuroscience and Physiology, Sahlgrenska Academy at the University of Gothenburg, Mölndal 431 80, Sweden; Comparative Medicine, Broad Institute of MIT and Harvard, Cambridge, MA 02142, USA; Department of Psychiatry and Neurochemistry, Institute of Neuroscience and Physiology, Sahlgrenska Academy at the University of Gothenburg, Mölndal 431 80, Sweden; Department of Psychiatry and Neurochemistry, Institute of Neuroscience and Physiology, Sahlgrenska Academy at the University of Gothenburg, Mölndal 431 80, Sweden; Comparative Medicine, Broad Institute of MIT and Harvard, Cambridge, MA 02142, USA; Clinical Microbiology Laboratory, Department of Pathology, Brigham and Women’s Hospital, Boston, MA 02115, USA; Clinical Microbiology Laboratory, Department of Pathology, Brigham and Women’s Hospital, Boston, MA 02115, USA; Clinical Microbiology Laboratory, Department of Pathology, Brigham and Women’s Hospital, Boston, MA 02115, USA; Clinical Microbiology Laboratory, Department of Pathology, Brigham and Women’s Hospital, Boston, MA 02115, USA; IQ Proteomics, Framingham, MA 01702, USA; IQ Proteomics, Framingham, MA 01702, USA; IQ Proteomics, Framingham, MA 01702, USA; IQ Proteomics, Framingham, MA 01702, USA; In Vivo Drug Metabolism and Pharmacokinetics, Charles River Labs, Worcester, MA 01605, USA; Clinical Microbiology Laboratory, Department of Pathology, Brigham and Women’s Hospital, Boston, MA 02115, USA; Massachusetts Host-Microbiome Center, Brigham and Women’s Hospital, Harvard Medical School, Boston, MA 02115, USA; McCance Center for Brain Health and Department of Neurology, Massachusetts General Hospital, Boston, MA 02114, USA; Department of Neurology, Harvard Medical School, Boston, MA 02115, USA; Program in Brain Health, Broad Institute of MIT and Harvard, Cambridge, MA 02142, USA; McCance Center for Brain Health and Department of Neurology, Massachusetts General Hospital, Boston, MA 02114, USA; Department of Neurology, Harvard Medical School, Boston, MA 02115, USA; Department of Psychiatry and Neurochemistry, Institute of Neuroscience and Physiology, Sahlgrenska Academy at the University of Gothenburg, Mölndal 431 80, Sweden; Clinical Neurochemistry Laboratory, Sahlgrenska University Hospital, Mölndal 431 80, Sweden; Department of Neurodegenerative Disease, UCL Queen Square Institute of Neurology, London WC1N 3BG, UK; UK Dementia Research Institute at UCL, London NW1 3BT, UK; Program in Brain Health, Broad Institute of MIT and Harvard, Cambridge, MA 02142, USA; McCance Center for Brain Health and Department of Neurology, Massachusetts General Hospital, Boston, MA 02114, USA; Department of Neurology, Harvard Medical School, Boston, MA 02115, USA

**Keywords:** biomarker, neurofilament, CSF, minocycline

## Abstract

Neurofilament light (NfL) concentration in CSF and blood serves as an important biomarker in neurology drug development. Changes in NfL are generally assumed to reflect changes in neuronal damage, while little is known about the clearance of NfL from biofluids. In a study of asymptomatic individuals at risk for prion disease, both blood and CSF NfL spiked in one participant following a 6-week course of minocycline, absent any other biomarker changes and without subsequent onset of symptoms. We subsequently observed high NfL after minocycline treatment in discarded clinical plasma samples from inpatients, in mouse plasma and in conditioned media from neuron–microglia co-cultures. The specificity and kinetics of NfL response lead us to hypothesize that minocycline does not cause or exacerbate neuronal damage, but instead affects NfL by inhibiting its clearance, posing a potential confounder for the interpretation of this important biomarker.

## Teaser

Gentile *et al.* report that treatment with the drug minocycline may cause neurofilament light concentration to rise in CSF and blood. This effect appears mediated by changes in clearance of the protein, rather than release from tissue, confounding this biomarker normally held to report on neuronal health.

## Graphical Abstract

**Graphical Abstract:**
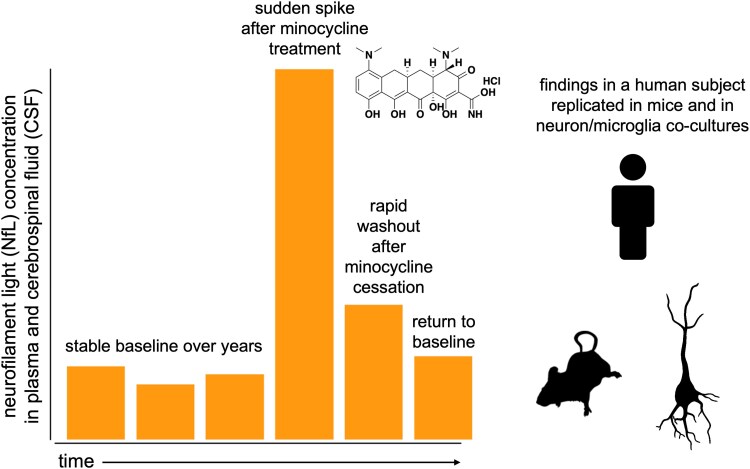

## Introduction

Neurofilament proteins light, medium and heavy (NfL, NfM and NfH) are components of an intermediate filament that structurally supports neurons.^1^ As neuronal damage causes leakage of these proteins from the cytosol into biofluids, NfL levels in CSF and blood are elevated, to various degrees, in virtually all neurological disorders.^2,3^ Although NfL is not diagnostically specific, it can potentially provide information on patient prognoses and treatment responses,^4^ for example, NfL is reduced by treatment in spinal muscular atrophy^5^ and multiple sclerosis.^4^ As a result, in the development of tofersen for *SOD1* ALS, NfL has been used both as an Accelerated Approval endpoint in symptomatic patients^6^ and as a criterion for randomization of pre-symptomatic patients to drug or placebo.^7^ Conversely, increased NfL has been cited as one reason for premature termination of the phase II trial of branaplam in Huntington’s disease.^8-10^

In principle, the concentration of any biomarker analyte is a function of both the rate of its production in tissue and release into fluids, as well as its catabolism and/or clearance from fluids. Indeed, while the above examples illustrate that NfL is widely used and interpreted as a marker of neurological insult, confounding factors are also recognized.^11,12^ For instance, NfL is inversely correlated with estimated glomerular filtration rate, suggesting removal through the kidneys, and with body mass index, perhaps due to dilution in a greater blood volume.^12^ A recent genome-wide association study demonstrated that a variant in *UMOD* implicated in kidney function affects plasma NfL levels, reinforcing a role for clearance pathways in steady state NfL levels,^13^ To date, however, factors affecting clearance of NfL within the CNS have not been identified.

Minocycline is a tetracycline antibiotic used clinically both for infections and for dermatologic conditions.^14-16^ It has been shown in rodents and in humans to suppress microglial activation and proliferation.^17-19^ This finding has led to clinical trials of minocycline in diverse neurologic indications; results have often been null or unfavorable,^20,21^ though minocycline was shown to reduce the risk of conversion from clinically isolated syndrome (CIS) to multiple sclerosis.^22^ In clinical trials of both traumatic brain injury (TBI) and CIS, randomization to minocycline was associated with a ∼3-fold increase in plasma NfL, which was interpreted as meaning that the drug may have contributed to neurodegeneration or was intrinsically neurotoxic.^19,23^

Prion disease exhibits a more dramatic elevation of NfL than that seen in perhaps any other neurological disorder.^24-26^ We and others have followed pre-symptomatic people at risk for genetic prion disease, however, and have found that plasma NfL increases in only a brief window prior to the onset of symptom,^26-29^ consistent with the rapid clinical course of prion disease.^30^ Here, we observed an NfL increase of 3.5-fold in plasma and 5.7-fold in CSF in an asymptomatic individual at risk for genetic prion disease following 6 weeks’ treatment with oral minocycline for a dermatologic indication. Other biomarkers remained normal, and proteomic analysis of CSF revealed that the spike was exquisitely specific to neurofilaments. NfL dropped nearly to normal levels 5 weeks after minocycline cessation, and the individual remained free of disease 2 years later. Plasma NfL in dermatology patients was not elevated above normal controls. Dramatically high plasma NfL (>500 pg/mL) was variably observed in some hospitalized individuals receiving minocycline. In mice, treatment with minocycline resulted in variable increases of 1.3- to 4.0-fold in plasma NfL, with complete wash-out 2 weeks after cessation. In neuron–microglia co-cultures, minocycline increased NfL concentration in conditioned media by 3.0-fold without any visually obvious impact on neuronal health. We hypothesize that minocycline does not cause or exacerbate neuronal damage, but instead impacts the clearance of NfL from biofluids, a potential confounder for interpretation of this biomarker.

## Materials and methods

### Ethical approvals

Human samples were collected through Mass General Brigham (MGB) Institutional Review Board (IRB) protocols 2015P000221, 2017P000214 and 2021P002185. Our prion cohort^27,29^ research participant provided written informed consent. Discarded plasma samples were collected under a waiver of consent approved by the MGB IRB. The four-digit hexadecimal IDs shown in the supplementary tables and supplementary figures were randomly generated and are not known to anyone outside the study group. Animal studies were performed under Broad Institute IACUC protocol 0162-05-17 and Charles River I026.

### Discarded plasma samples

Discarded plasma samples were collected through the Crimson Core Facility at Brigham and Women’s Hospital (‘Crimson Core’) from between September 2021 and March 2023. Monthly queries in Epic were used to update a list of patients age 18–89 meeting the following criteria in the prior 90-day period: (i) a dermatology encounter and a diagnosis of acne, rosacea, dermatitis, or atopic dermatitis/eczema and a prescription start for minocycline, isotretinoin, doxycycline, clindamycin, or azithromycin; or (ii) an annual physical exam encounter; or (iii) inpatients with an order for minocycline. The list of matching medical record numbers was then flagged for plasma pulls at the Crimson Core at the point of sample discard. Any lavender top K_2_-EDTA tube plasma sample from one of these individuals for which all clinical testing was completed and 48 h had passed was then pulled for inclusion in this study and labelled with a de-identified ID. Samples spent up to 8 h at room temperature and up to 2 days at 4°C before being frozen at −20°C for transient storage and then shipped on dry ice to the analysing laboratory for storage at −80°C. For individuals for whom plasma samples were obtained, sex, age, medication history, problem list and, where applicable, date of death were queried. Each individual was assigned a ‘zero’ date corresponding to the earliest date of any prescription start or blood sample collected. Sample dates, prescription start and end dates and death dates were then normalized to this zero. For analysis purposes, based on the duration of NfL elevation observed in the index case, patients were considered to be ‘on’ drug from the prescription start date through 7 days after prescription end date. For outpatient prescriptions with no end date specified, the prescription was considered active for 30 days. Upon thaw, plasma samples were re-centrifuged and supernatants were used for NfL analysis. Ages were binned in 5-year intervals. Keys linking samples to medical record numbers were destroyed and all identifiable data were deleted; as noted above, the four-digit hexadecimal IDs shown in the supplementary tables and supplementary figures were randomly generated and are not known to anyone outside the study group. The daily dose of drug was determined programmatically based on the mg of the formulation prescribed and the number of pills and frequency of use indicated in a free text field in Epic. For patients receiving minocycline, the 200 mg daily dose accounted for 52% of all patients queried (238/457) or 70% of those for whom dose was determined (238/340), including 10/10 for whom dose was determined and NfL data were collected in this study.

### Biomarker assays

NfL was determined on a Simple Plex Human NF-L Cartridge (Bio-Techne SPCKB-PS-002448) on a ProteinSimple Ella Automated Immunoassay System (Bio-Techne 600-100) according to the manufacturer’s instructions. Total tau was determined by ELISA (Fujirebio Innotest hTau AG 81579) according to the manufacturer’s instructions, with 450 nm absorbance read on a FLUOstar OPTIMA platereader (BMG Labtech). RT-QuIC was performed using the standard IQ-CSF protocol^31^ on a FLUOstar OPTIMA platereader (BMG Labtech) using SHaPrP90-230 produced in-house according to published procedures.^32^ We do not routinely measure blood T-tau in our cohort study because this biomarker exhibits relatively poor intra-individual reliability (test-retest CV = 38%).^27^

### Animals and drug treatment

All animals were wild-type C57BL/6N aged 6–20 weeks at study start from Charles River Laboratories. Mouse Studies 1 and 3 utilized mixed-sex animals (half male, half female) while mouse Studies 2 and 4 utilized females only. Animals were housed in groups of 4 on a 12/12 light/dark cycle with food and water *ad libitum*. Bleeds in the natural history study (Supplementary Fig. 2) and in minocycline treatment Studies 3 and 4 were performed submandibular, while those in minocycline treatment Studies 1 and 2 were performed via tail vein. Minocycline HCl (Gold Bio M-890-1) was made fresh daily in sterile saline and stored in the dark until intraperitoneal (i.p.) administration. Detailed parameters of each mouse study are compared in Supplementary Table 28.

### Sex as a biological variable

Our study examined male and female animals (see Animals and drug treatment), and similar findings are reported for both sexes. Supplementary Fig. 2 includes a natural history study of plasma NfL in mice, where any biological variability between sexes is shown.

### Human-induced pluripotent stem cell–derived cortical neurons and microglia

Human-induced pluripotent stem cell (hiPSC) from the commercially available cell line WTSIi015-A (Sigma) was used to derive cortical neurons and microglia. hiPSCs were cultured on Matrigel (Corning)-coated wells in mTeSR1+ medium (Stemcell Technologies). For the differentiation to neurons, no new neuronal induction was done, but previously frozen neuronal progenitor cells (NPCs) were utilized. The induction of NPCs from hiPSCs has been previously described.^33^ NPCs from induction day ∼30 were thawed for this project and cultured in laminin-coated wells in neural maintenance media (NMM) containing equal amounts of Dulbecco’s modified Eagle’s medium with F12 and L-Glutamax (DMEM/F12 L-Glutamax) and neurobasal medium supplemented with insulin, beta-mercaptoethanol (Thermo Fischer Scientific), non-essential amino acids (MEM-NEAA, Thermo Fisher Scientific), an antibiotic cocktail of penicillin and streptomycin (PEST, Hyclone, GE), sodium pyruvate (Sigma Aldrich), N2, B27 and GlutaMAX (all three from Thermo Fisher Scientific), in concentrations previously described (Fruhwuerth, 2023). Media were changed every other day, and NPCs were passaged at ∼80% confluency. After ∼2.5 weeks in culture, cells were passaged for a final seed-out on laminin-coated wells at a density of 5e4 cells/cm^2^. Cells were cultured for another week in NMM before hiPSC-derived microglia were added at a density of 20.000 cells/cm^2^ to create co-cultures. For the differentiation to hiPSC-derived microglia, a previously described protocol was followed.^33^ Briefly, hiPSCs are cultured on Matrigel (Corning) for about 1.5–2 weeks before embryonic body formation is initiated. Here, cells are dissociated with TrypLe Express (Thermo Fisher Scientific) and 4 × 10^6^ single cells are seeded into one well of an AggreWell 800 plate (Stemcell Technologies) in embryonic body medium consisting of mTeSR1+ supplemented with recombinant human bone morphogenic protein 4, recombinant human stem cell factor and recombinant human vascular endothelial growth factor 121, as well as Rock inhibitor (all from PeproTech). After 4 days, embryonic bodies were plated into a 6-well plate in hematopoietic medium (HM) consisting of X-VIVO 15 medium (Lonza) supplemented with beta-mercaptoethanol, GlutaMAX and PEST as well as human macrophage colony-stimulating factor and human interleukin-3 (both from PeproTech). After about 3–4 weeks with weekly media changes, primitive macrophage precursor cells can be harvested. Cells are counted, centrifuged and resuspended in co-culture media (CoM). CoM is NMM with addition of the growth factors Il-34 and GM-CSF at a concentration of 100 and 10 ng/mL, respectively. About 2.5e4 cells/cm^2^ are added to neuronal cultures and differentiated to microglia for 7–10 days before incubation with minocycline is started.

### Drug treatment of cell cultures

Cells were cultured in CoM until neuronal differentiation Day 60–65 and microglial differentiation Day 7–10 before addition of 25 µM minocycline (Sigma, #M9511–25 mg) to CoM. The C_max_ for 200 mg p.o. minocycline in humans was estimated at 3.1–3.6 mg/L,^34^ corresponding to 7–8 µM. The brain:plasma ratio of minocycline in rats was reported to be 0.35,^35^ but after chronic oral dosing of minocycline in drinking water in mice, brain concentrations up to 20 µM were reported.^36^ Without knowledge of the brain concentrations achieved in our mice or human patients, we chose a concentration of 25 µM, which did not affect cell viability (Supplementary Fig. 5) and which we hypothesized would be high enough to mirror any activity *in vivo*. Minocycline was dissolved in phosphate-buffered saline solution (DPBS, Gibco, #14190094) to a concentration of 5 mg/mL and stored in aliquots at −80°C until used for cell culture experiments. Minocycline treatment lasted for 6 days, followed by a wash-out period of 4 days. Media were collected at the start of minocycline treatment as well as with media change every other day until the end of the experiment. For image analysis, cells were fixed after 6 days of minocycline treatment (see details below under Immunocytochemistry and microscopy).

### Cell viability measurement

To assess cell viability after 6 days of minocycline treatment, we used the CyQuant TM XTT Cell Viability Assay (Invitrogen, X12223) following the manufacturer’s instructions. After 4 h of incubation, media were collected, and specific absorbance was measured using a TECAN Plate Reader. In this assay, higher specific absorbance indicates higher cell viability.

### NfL quantification in conditioned media

Every 2 days, the full volume of conditioned media was removed, centrifuged at 400*×g* at 6°C for 7 min, and supernatants were frozen at −80°C until analysis. NfL levels were measured at the Clinical Neurochemistry Laboratory at the University of Gothenburg, Sweden. After thawing, samples were prepared for measurement with HD-X ultra-sensitive single molecule array (Simoa) platform (Quanterix). NfL was measured using the Neurology 4-Plex Kit (Lot # 503864, Quanterix) following the manufacturer’s instructions.

### Immunocytochemistry and microscopy

For immunocytochemistry, cells were seeded in Ibidi-slides (Ibidi, #80806) at the same density as described above. After 6 days of treatment with or without minocycline, cells were washed with PBS and fixated with Histofix (Histolab, #01000) for 15 min. Cells were washed with Tris-buffered saline (TBS), permeabilized for 15 min in permeabilization buffer (0.3% Triton X-100 in TBS) and blocked with blocking buffer (5% goat serum in permeabilization buffer) for 1 h at room temperature. Cells were then incubated in primary antibody solution for Iba1 (1:500 in blocking buffer, Cell Signaling, #17198) and TuJ1 (1:500 in blocking buffer, Abcam, ab78078) overnight at 4°C. After a further wash with TBS, secondary antibody solution (Alexa-Fluor 568 goat anti-rabbit, Alexa-Fluor 488 goat anti-mouse) was added for 1 h at room temperature. After another wash with TBS, samples were incubated for 5 min with the cell nuclei stain DAPI in TBS (1:1000), followed by another wash with TBS. Finally, Ibidi-mounting media (Ibidi, #50001) was added and cells were visualized using a Nikon A1 inverted confocal microscope. Pictures presented are z-stacks covering 4 µm. For analysis of axonal depolymerization, z-stacks were merged for median intensity using Fiji ImageJ.^37^ Analysis followed a previously described method^38^: images were binarized with a localized Otsu threshold, such that axonal pixels are white and all other pixels are black. The Otsu threshold was calculated using the average 0.1% threshold from all control images within the same experiment, and this threshold was then applied to all images. The axonal microtubule depolymerization ratio was calculated as the ratio of pixels in depolymerized axons to those in intact axons, and this ratio was further normalized to the mean of control wells in each experiment. Three to five images per well were analysed.

### Transcriptomics

In mouse Study 1, 200 mg pieces of cortex were dissected from whole hemispheres frozen in RNAlater. Bulk RNA-seq was performed on cortex from three minocycline-treated animals euthanized with weight loss on Day 13, versus eight untreated control animals harvested on Day 16. RNA sequencing was performed by the Broad Institute Genomics Platform (Tru-Seq Strand Specific Large Insert RNA Sequencing) targeting 50 million read pairs. Transcript quantification was performed using Salmon^39^ on Terra.bio.

### Proteomics

Prior to proteomics analysis, CSF samples from the individual at risk for genetic prion disease were first pre-processed in a dedicated prion laboratory consistent with best practices.^40,41^ Analysis was then performed by IQ Proteomics (Framingham, MA) (see Supplementary Methods for details).

### Statistics, data and source code

All analyses were performed in R 4.2.0. Because NfL concentrations are non-normally distributed, they were visualized using medians and interquartile ranges and were compared using Wilcoxon tests. The dermatology cohort was analysed using log-linear regression: lm(log(nfl) ∼ drug + age). Because some inpatients had both ‘on’ and ‘off’ samples while others had both, inpatient data were compared using a partially paired Wilcoxon implemented in the R package robustrank. The volcano plot of TMT data in minocycline-treated mice was produced by grouping peptides by gene symbol using Empirical Brown’s Method^42^ (R Bioconductor) with subsequent Bonferroni correction. *P* values less than 0.05 were considered nominally significant. Transcriptomic analysis used DESeq2^43^ on Terra (Terra.bio) with default parameters and Benjamini–Hochberg control of false discovery rate. Genes were annotated as microglial if they had mean count > 0.1 in microglia and log2 fold change > 2 with nominal *P* < 0.05 compared to all other cell types based on mouse cortex 10X single cell data^44^ analysed in Loupe Browser. Source code and an analytical dataset sufficient to reproduce all figures and statistics in this manuscript are publicly available at https://github.com/ericminikel/mino.

## Results

An asymptomatic cohort study^27,29^ participant under age 50 harbouring a *PRNP* mutation exhibited a spike in NfL concentration in both plasma and CSF unaccompanied by any change in other fluid biomarkers of prion disease (Table 1). At this same study visit, this individual reported having just completed, 4 days earlier, a 6-week course of 200 mg/day oral minocycline for a dermatologic indication. NfL levels dropped substantially 5 weeks after minocycline cessation and were normal 1 year later. This individual has not developed symptoms of prion disease in >2 years of follow-up after the NfL spike. Based on plasma NfL increases in trials of minocycline in TBI and MS-CIS,^19,23^ we speculated that minocycline might have been the cause of the biofluid NfL increase in our study participant.

**Table 1:** Fluid biomarker values in a cohort study participant before and after taking minocycline

Visit	Plasma NfL (pg/mL)	CSF NfL (pg/mL)	CSF RT-QuIC	CSF T-tau (pg/mL)
Baseline 1	10.9	520	Negative (0/4)	188
Baseline 2	11.5	391	Negative (0/4)	176
Baseline 3	11.8	463	Negative (0/4)	169
4 days post	41.4	2633	Negative (0/4)	188
5 weeks post	13.7	959	Negative (0/4)	160
1 year post	5.8	591	Negative (0/4)	155

Baselines were taken over a >3 year period prior to minocycline treatment. ‘Post’ indicates time post cessation of minocycline. Parenthetical RT-QuIC values indicate positive replicates over total replicates.

**Figure 1:**
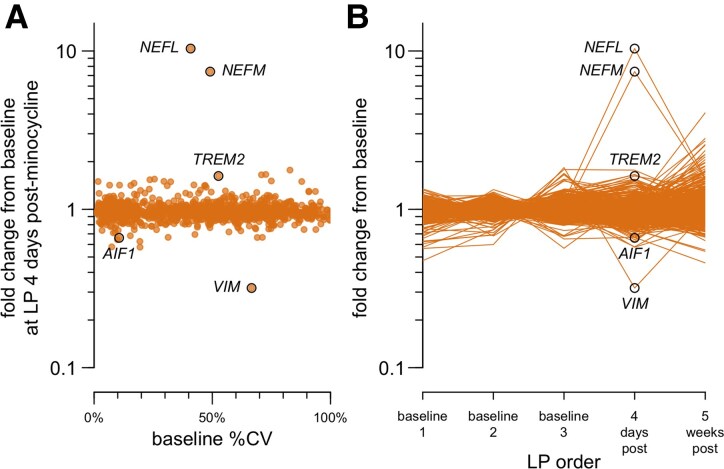
**Proteomic analysis of serial CSF samples from the minocycline-treated index case.** CSF samples collected by lumbar puncture (LP) were analysed by TMTs in technical duplicate in a single plex. Of the 6705 peptides quantified (Supplementary Table 1), 6588 with technical replicate coefficient of variation (CV) < 25% were grouped by gene symbol (note italicized gene symbols: *NEFL* = NfL, neurofilament light; *NEFM* = NfM, neurofilament medium) to yield relative protein abundance (Supplementary Table 2). Of the 1215 gene symbols encoding detected peptides, 1190 had a coefficient of variation (%CV) among the three baseline samples (baseline %CV) of <100% and were analysed here. (**A**) Coefficient of variation among the three baseline samples (baseline %CV) versus fold change at 4 days post-minocycline relative to the baseline mean. Each point represents one gene symbol. (**B**) Ratio of each sample’s abundance to the mean of the three baseline visits. Each point represents one gene symbol at one CSF sampling time point.

We performed proteomics using tandem mass tags (TMTs) on duplicate CSF samples from this individual’s first five serial lumbar punctures (Fig. 1). Of the 1190 proteins quantified, increases at 4 days post-minocycline were exquisitely specific to NfL (*NEFL*, one unique peptide) and NfM (*NEFM*, five unique peptides) (Fig. 1A; Supplementary Tables 1 and 2), with smaller changes in microglial proteins TREM2 and AIF1, and a reduction in intermediate filament VIM. By TMT, all of these proteins returned to their baseline levels within 5 weeks (Fig. 1B). Replication of the increase in neurofilament by this mass spectrometry method, orthogonal to the immunoassay used initially (Table 1), rules out a direct assay interference effect.

**Figure 2:**
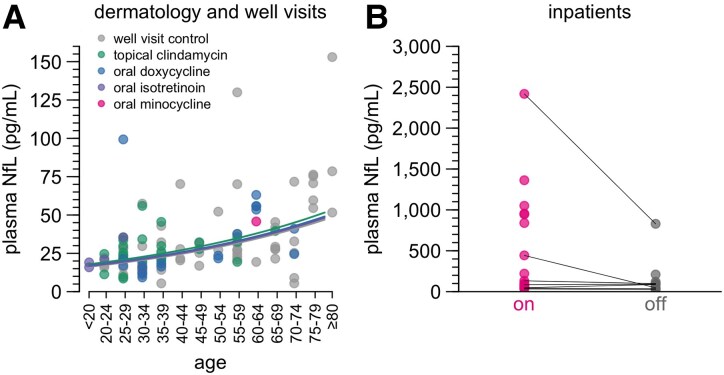
**Plasma NfL levels in discarded plasma cohorts.** (**A**) Plasma NfL in control patients with a recent well visit (grey) or with a recent dermatology encounter, diagnosis of acne, rosacea, dermatitis or eczema and a recent prescription start for topical clindamycin, oral doxycycline, oral isotretinoin or oral minocycline. For detailed inclusion criteria, see Materials and methods. Points represent individual patients; where multiple blood samples were available for one patient, the mean plasma NfL value is plotted. Curves represent log-linear regression best fits for each group with *n* > 1. *n* = 113 individuals in total; for individual values and model fits, see Supplementary Tables 4 and 5. (**B**) Plasma NfL in inpatients prescribed minocycline, *n* = 215 samples from *n* = 20 individuals. Points represent the mean on and mean off values for each patient, with thin lines connecting on/off means for the same patient. *P* = 0.059, two-sided partially paired Wilcoxon test. Individual NfL trajectories and times on/off drug for each inpatient are shown in Supplementary Fig. 1. Inpatient details are provided in Supplementary Tables 6–8. Route of administration was oral (*n* = 12), intravenous (*n* = 6) or both at various times (*n* = 2). Dose was 200 mg/day in all cases where it could be determined (see Materials and methods).

Although our index case harbours a pathogenic *PRNP* mutation, the lack of manifest disease 2 years after the NfL spike led us to wonder whether minocycline would elicit an increase in biofluid NfL levels in individuals lacking any predisposition to neurodegeneration. We therefore collected discarded plasma samples from dermatology patients with diagnoses of acne, rosacea, eczema or dermatitis—reasoning that such patients are generally otherwise healthy—who had a new prescription start in the past 30 days (Fig. 2A; Supplementary Fig. 1 and Tables 3 and 4) and had an order for complete blood count to allow collection of the excess whole blood after completion of clinical testing. We also considered well visit controls unselected for starting any medications. However, as blood testing is not routinely ordered in outpatient dermatology visits, our sample collection was inherently limited to individuals who happened to have had a blood draw for any other reason around the time of their dermatology visit.

Samples from the entire discarded plasma cohort generally exhibited higher NfL values than seen in our own prion disease cohort study or in established reference ranges^45^ (Fig. 2A). This finding was observed across all groups including well visit controls and thus may be pre-analytical in nature, though NfL is reported to be robust to pre-analytical variables,^3,46,47^ and we were unable to identify anything anomalous in the sample handling protocol (Materials and methods). The higher-than-typical readings corresponded to higher baselines across all age groups, with a mean plasma NfL level of 16.7 pg/mL at 18 years of age. The relationship between NfL and age (+1.7% per year of life, log-linear regression; Supplementary Table 5) was not higher than in previous reports.^45,46,48^ Unfortunately, over an 18 month window for prospective sample collection, we obtained plasma from only one dermatology patient with a recent oral minocycline start; this one individual’s plasma NfL was not anomalous relative to other samples in this collection. NfL levels in dermatology patients receiving oral doxycycline or isotretinoin, or topical clindamycin, were not significantly different from well visit controls, arguing that these dermatological conditions do not themselves increase NfL (Fig. 2A).

We also collected discarded plasma samples from hospital inpatients who received minocycline, usually a 200 mg daily dose (see Materials and methods). As these patients had disparate diagnoses, reasons for hospitalization (Supplementary Table 6) and reasons for minocycline administration, we used these individuals as their own controls, comparing plasma NfL in blood draws collected while on and off minocycline (Fig. 2B). Samples from patients receiving minocycline were not significantly higher than during periods of time during which they were not receiving the drug (*P* = 0.059, partially paired Wilcoxon test). A subset of patients demonstrated extremely high (>500 pg/mL) NfL while receiving minocycline, a level not observed in any off-minocycline samples, except for one individual who dropped from a mean of 2418 pg/mL while receiving minocycline, to 830 pg/mL after completing minocycline therapy. Within our inpatient cohort, no specific clinical covariates were identified with individuals with ultra-high NfL while receiving minocycline, for instance, not all had neurological conditions (Supplementary Tables 6–8).

To fill gaps in the clinical cohorts to power specific questions of NfL levels relative to minocycline exposure, we established a rodent model to determine minocycline’s effects on plasma NfL. To establish a baseline, we serially sampled plasma and measured NfL in C57BL/6N mice aged 6–22 weeks (*n* = 272 samples total; Supplementary Fig. 2A and Table 9). Plasma NfL is both higher and more variable in male than in female mice (mean 167 versus 86 ng/mL, mean CV 97% versus 46% across all samples; Supplementary Fig. S2B and C and Tables 10 and 11). In males, inter-animal variability was higher than within-animal variability over time (CV = 62% versus 48%; Supplementary Fig. 2D and E), but this was not the case in females (CV = 23% between-animal and 37% within-animal; Supplementary Fig. 2D and E). This finding suggested that mouse studies with plasma NfL as the primary outcome are better powered with female mice than male or mixed-sex cohorts. In addition, study designs comparing animals to their own baseline do not necessarily have any advantage over study designs comparing animals to one another.

**Figure 3:**
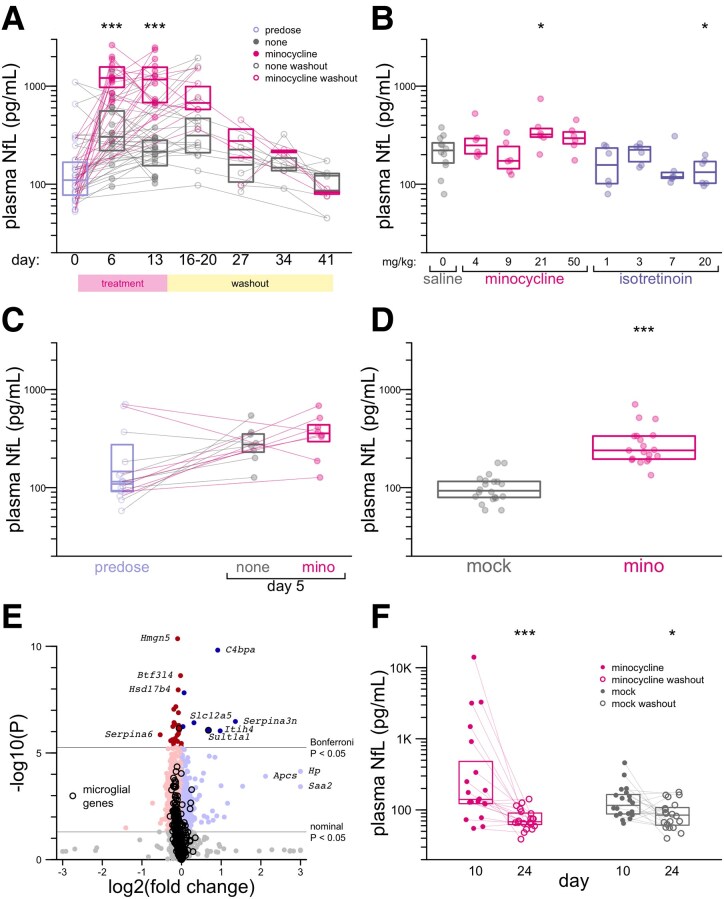
**Neurofilament in mice treated with minocycline.** (**A**) Plasma NfL values in mouse Study 1, with 50 mg/kg minocycline. See text for details. Points represent individual animal–time point combinations. (**B**) Plasma NfL values in mouse Study 2, doses as indicated. Points represent individual animals. (**C**) Plasma NfL values in mouse Study 3, with 50 mg/kg minocycline. Points represent individual animals. (**D**) Plasma NfL values in mouse Study 4, after 5 days of 50 mg/kg minocycline. Points represent individual animal–time point combinations. (**E**) TMTs on whole brain hemispheres of *n* = 9 minocycline versus *n* = 9 mock-treated mice in mouse Study 4, sacrificed at Day 5. Each point represents one gene symbol (*n* = 9051 proteins quantified). *P* values are obtained by Empirical Brown’s method with Bonferroni correction. (**F**) Plasma NfL values in mouse Study 4, after 10 days of 50 mg/kg minocycline and after 14 subsequent days of wash-out (no dosing). Points represent individual animal–time point combinations. In **A–D** and **F**, two-sided Wilcoxon tests are used; *P < 0.05, **P < 0.01, ***P < 0.001.

Minocycline has a shorter half-life in rodents than in humans, such that a 50 mg/kg dose in mice is estimated to provide exposure similar to the common clinically used dose of minocycline, 200 mg daily, which corresponds to ∼3 mg/kg for a 70 kg human.^34,49,50^ This factor is consistent with allometric (body surface area) scaling used for many small molecule drugs.^51^ We treated *n* = 16 mixed-sex mice per arm with once daily 50 mg/kg minocycline i.p., a route of administration for which pharmacokinetics in mice have been characterized.^49,50^ After 6 days of dosing, plasma NfL was 4.0 times higher in minocycline-treated mice than untreated mice (median 1211 versus 304 pg/mL, *P* = 1.8e-6, Wilcoxon test; Fig. 3A; Supplementary Tables 12–14). This difference grew further by 13 days (median 1169 versus 214 pg/mL, *P* = 3.6e-6; Fig. 3A). However, this dose was not well-tolerated over this length of time: by day 10, the average minocycline-treated mouse had lost 6% body weight, and some began to require euthanasia (Supplementary Fig. 3A and Tables 15 and 16). Dosing was therefore discontinued after day 15; plasma NfL in the formerly minocycline-treated animals then dropped rapidly and was not significantly higher than the untreated animals thereafter (Fig. 3A). Bulk RNA-seq on the cortex of minocycline-treated versus untreated animals identified several downregulated microglial genes but also a large number of apparently non-specific hits that may reflect cellular distress or death (Supplementary Fig. 3B and Table 17).

We sought to determine whether a better-tolerated dose of minocycline might also raise NfL. We also sought to determine the effect on plasma NfL of isotretinoin which, like minocycline, suppresses microglial response to LPS challenge *in vivo*.^17^ We therefore conducted a 6-day, 4-point dose-response study for each compound with *n* = 6 female animals per dose and *n* = 12 in the saline group. In this study, the original 50 mg/kg dose of minocycline yielded only a non-significant 1.3-fold increase in plasma NfL compared to saline animals (median 294 versus 221 pg/mL, *P* = 0.12, Wilcoxon test; Fig. 3B; Supplementary Tables 18 and 19). Isotretinoin did not increase NfL (Fig. 3B). A repeat study of 5 days of 50 mg/kg dosing in *n* = 8 mixed-sex animals per arm yielded similarly equivocal results (1.3-fold increase, median 358 versus 276 pg/mL, *P* = 0.40, Wilcoxon test; Fig. 3C; Supplementary Tables 20 and 21).

Incorporating learnings from our study of the natural history of plasma NfL in mice (Supplementary Fig. 2), we conducted a fourth mouse study using *n* = 20 female animals per group (Fig. 3D; Supplementary Tables 22 and 23). After 5 days of 50 mg/kg dosing, plasma NfL in minocycline-treated animals was 2.6-fold higher than untreated animals (median 240 versus 93 pg/mL, *P* = 1.6e-7, Wilcoxon test; Fig. 3D). Dosing for this duration was reasonably well-tolerated, although minocycline animals did lose 0.5% body weight on average over 5 days, a significant difference from the 2.4% weight gain in control animals (*P* = 4.4e-5, *t*-test; Supplementary Tables 24 and 25). Proteomic changes in the brains of mice that received minocycline for 5 days were relatively quiet (Fig. 3E; Supplementary Table 26). Microglial genes (Supplementary Table 27) were disproportionately downregulated, but the vast majority were well below the significance threshold after multiple testing correction. Top significantly altered transcripts included complement-binding protein (*C4bpa*), and a microglia-expressed sulfotransferase involved in xenobiotic metabolism (*Sult1a1*) but did not collectively point to any one obvious pathway. Parenchymal expression of neurofilament itself was not increased. Markers of neuroinflammation (e.g. *Gfap*) and neurological insult (e.g. *Mapt*) were unaltered. In mice that received 10 days of minocycline followed by 14 days without dosing, median NfL dropped by 51% (from 140.5 to 62.8 pg/mL, *P* = 9.3e-5, Wilcoxon test), although a 27% decrease was also observed in mock-treated animals (84.6 versus 116.0 pg/mL, *P* = 0.016, Wilcoxon test; Fig. 3F). Overall, differences in magnitude of effect across the four mouse studies could not be readily attributed to any one experimental variable (Supplementary Table 28).

**Figure 4:**
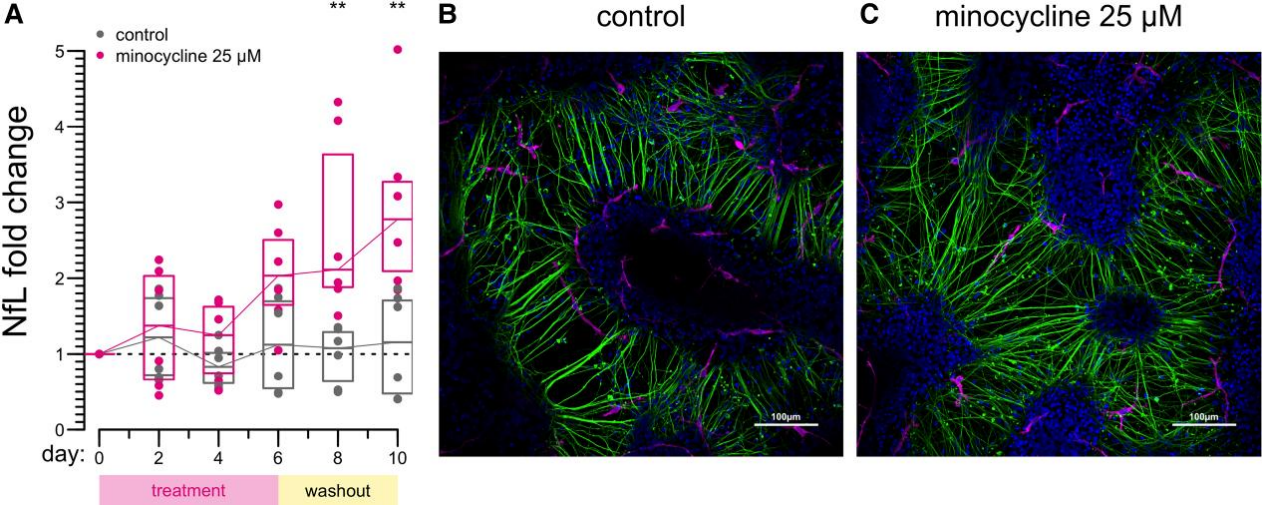
**Neurofilament in conditioned media of neuron–microglia co-cultures.** (**A**) NfL concentration in conditioned media of neuron–microglia co-cultures treated with 25 µM minocycline or no treatment. Points represent the *n* = 6 biological replicates (culture wells) per condition, followed serially over 10 days; every 2 days, media were removed and analysed for NfL, and new media were added. Boxes represent medians and interquartile ranges. **P < 0.01 by two-sided Wilcoxon test. Raw and summarized values are provided in Supplementary Tables 29 and 30. (**B** and **C**) Representative images of control (**B**) and minocycline-treated (**C**) co-cultures. Blue: DAPI; green: TuJ1; Magenta: Iba1. Separate TuJ1 and Iba1 channels are provided in Supplementary Fig. 4.

We also sought to test whether minocycline might impact NfL concentration in conditioned media in a neuron–microglia co-culture system. After 65 days of differentiating cortical neurons from iPSC and 10 days of differentiating microglia into this system (see Materials and methods), we began treatment with 25 µM minocycline, a concentration above the ∼7–8 µM C_max_ in humans taking 200 mg minocycline daily (see Materials and methods), and thus expected to be active, while not affecting cell viability (Supplementary Fig. 5). Conditioned media were completely removed every 2 days and subjected to NfL measurements, while new media were added. After 6 days of minocycline treatment, NfL increased by 2.0-fold from the baseline at day 0 (*P* = 0.065 versus no treatment control wells). After minocycline was withdrawn, NfL continued to rise, reaching a 3.0-fold increase at day 10 (4 days post-withdrawal; *P* = 0.0043 versus control wells; Fig. 4A; Supplementary Tables 29 and 30). Imaging and cell viability measurements of the co-cultures revealed no obvious difference in health between control and minocycline-treated cells (Fig. 4B and C; Supplementary Figs. S4 and S5), and quantification of axonal depolymerization likewise revealed no significant difference (Supplementary Fig. 6 and Tables 31 and 32).

## Discussion

Increases in NfL are generally assumed to reflect neuronal distress or damage. Prior studies where randomization to minocycline resulted in an increase in plasma NfL were interpreted as suggesting that minocycline exacerbated neurodegeneration, or was to some degree intrinsically neurotoxic.^19,23^ Correspondingly, low NfL has been interpreted as a neuroprotective signal.^52^ Several observations here suggest that minocycline may be capable of modulating NfL via a neuronal health-independent mechanism.

First, both CSF and plasma NfL increased dramatically after 6 weeks’ minocycline treatment in a healthy research study participant. We likewise observed, variably, increases in NfL in plasma of wild-type, unchallenged mice treated with minocycline and in conditioned media of co-cultured neurons and microglia.

Our research participant harboured a genetic predisposition to prion disease, but, other than NfL, showed no biomarker evidence of disease process. Both the magnitude of this individual’s NfL increase—far beyond levels ever observed in prodromal genetic prion disease^26,28,29^ and indeed, higher than many symptomatic genetic prion disease patients,^26,53^ and the lack of disease onset 2 years later, argue that this spike does not correspond to the apparently very brief prodromal window in genetic prion disease. This individual also carried a dermatologic diagnosis which was the indication for minocycline treatment, but our analysis of discarded plasma from dermatology patients suggests that dermatologic conditions themselves are not a likely cause of elevated NfL. This individual took a 200 mg/day dose of minocycline, the most commonly prescribed clinical dose. If minocycline were so neurotoxic at this dose as to cause the magnitude of NfL increase observed here, one might expect this neurotoxicity to exhibit outward phenotypic manifestations, inconsistent with the relatively clean safety record and widespread clinical use of minocycline.

Second, the increase in NfL was highly specific. Other than a slight perturbation in microglial markers TREM2 and AIF1 and a decrease in intermediate filament VIM, no proteins other than NfL and NfM were altered in this individual’s CSF. If minocycline caused or exacerbated neurodegeneration, one might expect CSF T-tau to increase, which it did not. Similarly, in mice with elevated plasma NfL after 5 days of minocycline treatment, we observed a relatively quiet proteomic profile in brain parenchyma.

Third, the kinetics of NfL wash-out were rapid. In our study participant, NfL had returned almost to normal levels by 5 weeks after minocycline cessation. In two different mouse studies, we observed complete or nearly complete return to normal plasma NfL within 2 weeks of withdrawing minocycline. Spikes in plasma NfL due to acute neuronal insults are reported to take more than 12 weeks to clear.^19,54,55^

At the same time, our study has important limitations and many of the data collected here are inconsistent. We observed large and significant increases in NfL in only two of the four studies of minocycline treatment in mice. Despite using the same dose (50 mg/kg/day) over similar dosing duration (5–6 days) and generating directionally consistent results, the other two studies saw small and non-significant NfL increases. We were able to ascertain only one dermatology patient on minocycline, leaving us unable to determine whether minocycline increases NfL in this population. Moreover, while dermatology patients are often otherwise healthy, we were unable to rule out confounding diagnoses in this population. NfL levels were extremely high in a subset of hospitalized patients taking minocycline, but not in others, and overall, any difference in plasma NfL between on-drug and off-drug time points was non-significant. Most of these inpatients were on only short courses of minocycline, and we counted them as ‘on’ drug from the first day of treatment.

Our comparison of plasma NfL levels observed in our mouse studies is based on a time point following 5–6 days of treatment. In contrast, our research participant had been taking minocycline for 6 weeks, and increases in plasma NfL in TBI and MS-CIS were observed after 12 weeks. One possible explanation for our discordant findings would be that if minocycline inhibits the clearance or catabolism of NfL, then NfL concentration might increase rapidly (<1 week) in humans or mice that are releasing NfL rapidly, while it might rise much more slowly in humans releasing NfL at lower rates. CSF NfL was only modestly increased in *Grn* KO mice and was not increased in *Trem2* KO mice, but was dramatically increased in *Grn/Trem2* double KO mice,^56^ consistent with a ‘two-hit’ hypothesis where the effect of inhibiting microglial clearance of NfL is magnified when there is also a perturbation that increases the rate of NfL release. Our hypothesis would also be consistent with data from Alzheimer’s patients indicating an inverse correlation between microglial activation and NfL.^52^

Based on our results, we hypothesize that microglia play a role in clearing NfL and that minocycline inhibits this clearance, leading to an increase in CSF and plasma NfL concentration without causing neurodegeneration. At this time, however, this is only a hypothesis. It is not possible to make definitive conclusions, because our study has very prominent limitations. We possess detailed data on only one human research participant, and while our data argue against dermatological conditions themselves affecting NfL, we are unable to categorically rule out all other causes for increased NfL. Our data on dermatology patients and inpatients were collected only opportunistically, leading to incomplete clinical pictures, inconsistent numbers of days on drug and sporadic plasma sampling. There appear to be pre-analytical variables in our discarded plasma samples that we do not fully understand. We did not implement a pharmacokinetic assay to determine drug concentration in the human plasma samples but instead relied on medical records of prescriptions and drug administrations; the actual drug exposure levels in patients are unknown. The poor pharmacokinetics of minocycline in rodents compelled us to administer minocycline at a dose ∼16 times the typical clinical dose. This high dose was not tolerated for 10 days and even caused slight but significant weight loss by 5 days. Although this dose was not obviously toxic at 5–6 days, a time point when we already observed elevated plasma NfL, there remains doubt as to the degree to which toxicity may have contributed to the findings. Moreover, the massive variability in plasma NfL in mice means that some of our mouse studies may have been underpowered, and we do not know how much a lack of power versus genuine heterogeneity explains our discordant results. Our cell culture studies were performed at a concentration above the C_max_ reported in human plasma; however, only limited data on the pharmacokinetics of minocycline in brain are available. One study^35^ found that after a single dose in rats, the brain:plasma ratio was 0.35, while another study reported brain concentrations up to 20 µM in chronically treated mice.^36^ We cannot be certain that the concentration of minocycline used in cell culture accurately reflects concentrations achieved *in vivo*.

While the evidence here is very preliminary, the implications of this hypothesis could be important. Given the pivotal role that NfL has begun to play in neurological clinical trials, the possibility that drugs—and perhaps also genetic, environmental or other factors—might be capable of altering NfL concentration by a means other than causing or alleviating neurological insult, merits consideration.

## Supplementary Material

fcaf175_Supplementary_Data

## Data Availability

Data and source code are publicly available at https://github.com/ericminikel/mino.
