# Supplementary material for: Evidence that minocycline treatment confounds the interpretation of neurofilament as a biomarker
Source: Brain Commun. 2025 May 23;7(3):fcaf175. doi: 10.1093/braincomms/fcaf175 (PMC12100619; doi:10.1093/braincomms/fcaf175)
Supplement: fcaf175_Supplementary_Data [file fcaf175_supplementary_data.zip › supplementary-material.pdf]

SUPPLEMENT

Evidence that minocycline treatment confounds the interpretation of neurofilament as a biomarker

Gentile et al, 2024

Supplementary Tables

Supplementary Tables are provided as a separate Excel file.

Supplementary Methods

Sample processing for proteomics - CSF samples. Prior to proteomics analysis, CSF samples from the individual at risk for genetic prion disease were first pre-processed in a dedicated prion laboratory consistent with best practices[1,2]. 500 µL of each CSF sample was transferred to a depletion column to remove abundant plasma proteins (High-Select Top14 Abundant Protein Midi Spin Columns, Pierce), inverted several times to resuspend the depletion resin, then incubated on a rotisserie for 30 minutes at room temperature. The unbound fraction was retrieved by snapping off the column bottom end-cap, placing each column in a clean 15-mL tube and spinning at 1000 *g* for 3 minutes. Sodium dodecyl sulfate (SDS) was added to a final concentration of 2% (v/v), and dithiothreitol (DTT) to a final concentration of 5mM. After incubation at 37°C for 30 minutes samples were cooled to room temperature, treated with iodoacetamide (IAA; to 15mM final), and incubated in the dark for 1 hour at room temperature. Alkylation was quenched by adding DTT to 10mM final concentration. A protein pellet was then isolated by methanol chloroform precipitation, air dried, denatured in 8 M guanidine hydrochloride (GdnHCl) for 30 minutes, then diluted with 100mM EPPS buffer pH 8.1. Digestion was then performed with LysC (Promega V1671 at 1:50 enzyme:protein) overnight at room temperature followed by trypsin (Promega V5113 at 1:50 enzyme:protein) for 37°C for 6 hours.

Sample processing for proteomics - brain samples. Brains from uninfected mice were first homogenized at 10% wt/vol in 0.2% CHAPS as described[3] and homogenates were frozen at -80°C. Upon thaw, homogenates were treated with 4 M urea + 1% SDS + 100 mM EPPS pH 8.0 + HALT protease and phosphatase inhibitors (Thermo Fisher Scientific) and bead beaten at 2°C using a Precellys Evolution homogenizer for a total of 3 cycles: 10 second intervals at 7200 rpm with 60 second pauses. Protein concentration in the lysates were quantified using the Pierce micro-BCA assay (Thermo Fisher Scientific). Protein reduction was performed for 60 min at 25°C with 5 mM dithiothreitol (DTT). Free thiol containing cysteines were alkylated with 15 mM iodoacetamide and quenched with further addition of DTT to a final 10 mM prior to protein purification. The single-pot, solid-phase-enhanced, sample preparation (SP3) protocol[4,5] was applied to purify protein across all samples in these studies. Briefly, the magnetic beads were prepared by combining E3 and E7 Sera-Mag Carboxylate-Modified Magnetic SpeedBeads (1:1; vol:vol) (Cytiva). The beads were washed three times with HPLC H2O prior to resuspension in lysis buffer (1% SDS, 100 mM EPPS, pH 8.0) for a final bead slurry concentration of 50 µg/µL.

Approximately 20 µL of the pre-washed bead slurry and 75 µL 100% ethanol was added to each 30 µL sample containing 50 µg of protein (4M urea, 1% SDS, 100 mM EPPS pH 8.0). Proteins were allowed to bind and incubate with the bead slurry for 15 min at 25°C with periodic gentle vortexing. Following protein binding, the supernatant was discarded, and the bead-captured proteins were washed three times with 80% ethanol and air dried. The purified protein-captured beads were resuspended in 100 mM EPPS, pH 8.0. Approximately 50 µg of protein/sample was digested at 25°C for 12 hours with lysyl endopeptidase (LysC, Wako Chemicals USA) at a 1:12.5 (w/w) protease:protein ratio. Following LysC digestion, the peptides were digested with trypsin at 37 C for 8 hours (Promega) at a 1:25 (w/w) protease:protein ratio. The digested peptides (supernatant) were purified and separated from the magnetic beads. Three post-digestion washes were performed to wash and ensure elution of all peptides from the beads with the following buffers: 100 mM EPPS pH 8.0, 0.2% formic acid, and 80% acetonitrile/0.1% formic acid. With every wash and elution, the supernatant was transferred and combined with the initial supernatant and dried to completion by vacuum centrifugation. The percentage of missed cleavages was monitored across 4 random samples to ensure optimal digestion efficiency prior to isobaric labeling.

TMT labeling and fractionation - CSF samples. Following protein digestion with LysC and trypsin, the percentage of missed cleavages was monitored across four random samples, desalted, and analyzed by mass spectrometry to ensure optimal digestion efficiency prior to isobaric labeling. Isobaric labeling of peptides was performed with the 10-plex tandem mass tag (TMT) reagents (Thermo Fisher Scientific). The TMT10 reagents (120 µg per channel) were added to all samples and incubated for 2 hours at 25° C. A small portion (5%) of each sample was mixed together, desalted via Empore-C18 StageTip, and analyzed via LC-MS to check TMT labeling efficiency and loading ratio. All labeling reactions were quenched with hydroxylamine (0.5% final) and acidified with trifluoroacetic acid (2% final). The ten samples were mixed according to total summed TMT signal observed in the labeling efficiency check such that all samples have equal loading. The pooled TMT10 labeled peptide mix was desalted with a 50 mg tC18 Sep-Pak (Waters) and dried by vacuum centrifugation. The desalted, pooled TMT labeled peptides were fractionated using a high pH reverse-phase peptide fractionation kit (Pierce) into seven total fractions (10%, 15%, 17.5%, 20%, 30%, 50% and 70% acetonitrile in 0.1% triethylamine) and vacuum dried. The fractions were desalted on C18 StageTips and analyzed using a 180min SPS-MS3 method on an Orbitrap Eclipse instrument.

TMT labeling and fractionation - brain samples. All peptides (50 µg/sample) were resolubilized in 20 µL 500 mM EPPS, pH 8.0. Isobaric labeling of peptides was performed with the 18-plex tandem mass tag (TMT) reagents (Thermo Fisher Scientific). The TMTPro reagents (5 mg) were resuspended in 100 µL anhydrous acetonitrile (ACN) and 5 µL (200 µg) was added to all of the peptides and incubated for 2 hours at 25° C. A small portion (1%) of each sample was mixed together, desalted via C18 StageTip, and analyzed via LC-MS to check TMT labeling efficiency and loading ratio. All labeling reactions were quenched with hydroxylamine (0.5% final) and acidified with trifluoroacetic acid (2% final). The 18 channels were mixed according to total summed TMT signal observed in the labeling efficiency check such that all samples have

equal loading. The final TMT mixed sample was desalted with a 50 mg tC18 Sep-Pak (Waters) and dried by vacuum centrifugation. Approximately 100 µg of peptide mix was subjected to orthogonal basic-pH reverse phase fractionation on a 3x150 mm column packed with 1.9 µm Poroshell C18 material (Agilent, Santa Clara, CA), utilizing a 45 min linear gradient from 86% buffer A (5% acetonitrile in 10 mM ammonium bicarbonate, pH 8) to 42% buffer B (90% acetonitrile in 10 mM ammonium bicarbonate, pH 8) at a flow rate of 0.3 ml/min. Ninety six fractions were collected and consolidated into 24 total fractions, acidified with formic acid and vacuum dried. The fractions were resuspended in 0.2% formic acid, desalted on StageTips and vacuum dried. Peptides were reconstituted in 5% formic acid + 5% acetonitrile for LC-MS3 analysis.

Mass spectrometry analysis. All mass spectra were acquired on an Orbitrap instrument (Eclipse for CSF samples, Lumos for brain) coupled to an EASY nanoLC-1200 (Thermo Fisher) liquid chromatography system. Peptides (approximately 1 µg for CSF, 2 µg for brain) were loaded on a 75 µm capillary column packed in-house with Sepax GP-C18 resin (1.8 µm, 150 Å, Sepax) to a final length of 40 cm (CSF) or 35 cm (brain). Peptides for total protein analysis were separated using a 180 minute (CSF) or 90 minute (brain) linear gradient from 15% (CSF) or 14% (brain) to 40% acetonitrile in 0.1% formic acid. The mass spectrometer was operated in a data dependent mode. The scan sequence began with FTMS1 spectra (resolution = 120,000; mass range of 350-1400 *m/z*; max injection time of 10 ms; AGC target of 5e5; dynamic exclusion for 75 seconds (CSF) or 60 seconds (brain) with a +/- 10 ppm window). The most intense precursor ions were selected for ITMS2 analysis via collisional-induced dissociation (CID) in the ion trap (normalized collision energy (NCE) = 35; max injection time = 35ms; isolation window of 0.7 Da; AGC target of 1e4) within a 2 second window. A real-time search approach[6] was utilized during data acquisition to only trigger quantitative spectra on high-confidence peptide identifications. Online spectral identification was accomplished via a custom software client that monitors spectral acquisition though a vendor supplied instrument application programming interface and assigns peptide sequences through a probabilistic model, in real-time. The RTS client utilized a peptide database composed of *in-silico* predicted species-specific tryptic peptides. All mass spectra were converted to mzXML using a modified version of ReAdW.exe. MS/MS spectra were searched against a concatenated Uniprot species-specific protein database containing common contaminants (forward + reverse sequences) using the SEQUEST algorithm[7]. Database search criteria are as follows: fully tryptic with two missed cleavages; a precursor mass tolerance of 50 ppm and a fragment ion tolerance of 1 Da for peptides; oxidation of methionine (15.9949 Da) was set as a differential modification. Static modifications were iodoacetamide on cysteines (57.02146) and TMT on lysines and N-termini of peptides (CSF: TMT10: 229.1629 Da; brain: TMT18: 304.2071). Peptide-spectrum matches were filtered using linear discriminant analysis[8] and adjusted to a 1% peptide false discovery rate (FDR)[9] and collapsed further to a final 1.0% protein-level FDR. Proteins were quantified by summing the total reporter intensities across all matching PSMs.

Supplementary Figures

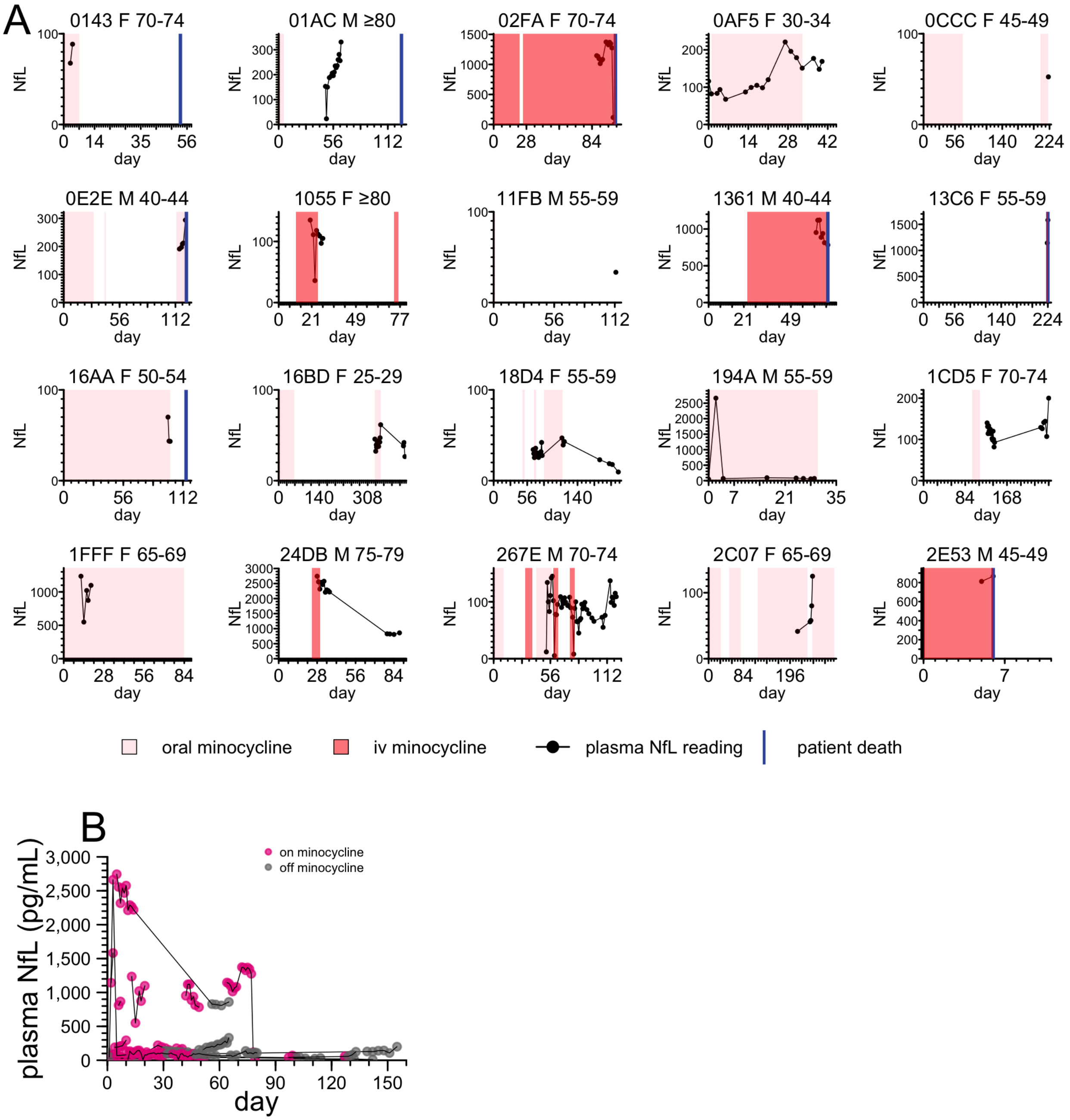

Supplementary Figure 1. Individual inpatient NfL trajectories and minocycline treatment. *This plot depicts the underlying data that are summarized in Figure 2B.* ***A)*** *Individual NfL trajectories overlaid with times of minocycline administration and, where applicable, day of death. Shown above each panel is a de-identified patient ID, which matches diagnosis and drug indication details shown in Supplementary Table 6, and the*

patient's sex and age. As noted in Methods, the 4-digit hexadecimal IDs used above were randomly generated and are not known to anyone outside the study group. Day 0 is set to the first date on which either a prescription began or a blood sample was collected. ***B)*** *The same trajectories shown overlaid in a single plot. Points represent individual blood samples, thin black lines connect serial samples from the same patient. Magenta points are timepoints when on drug and following at least 7 continuous days on drug, all other points are gray.*

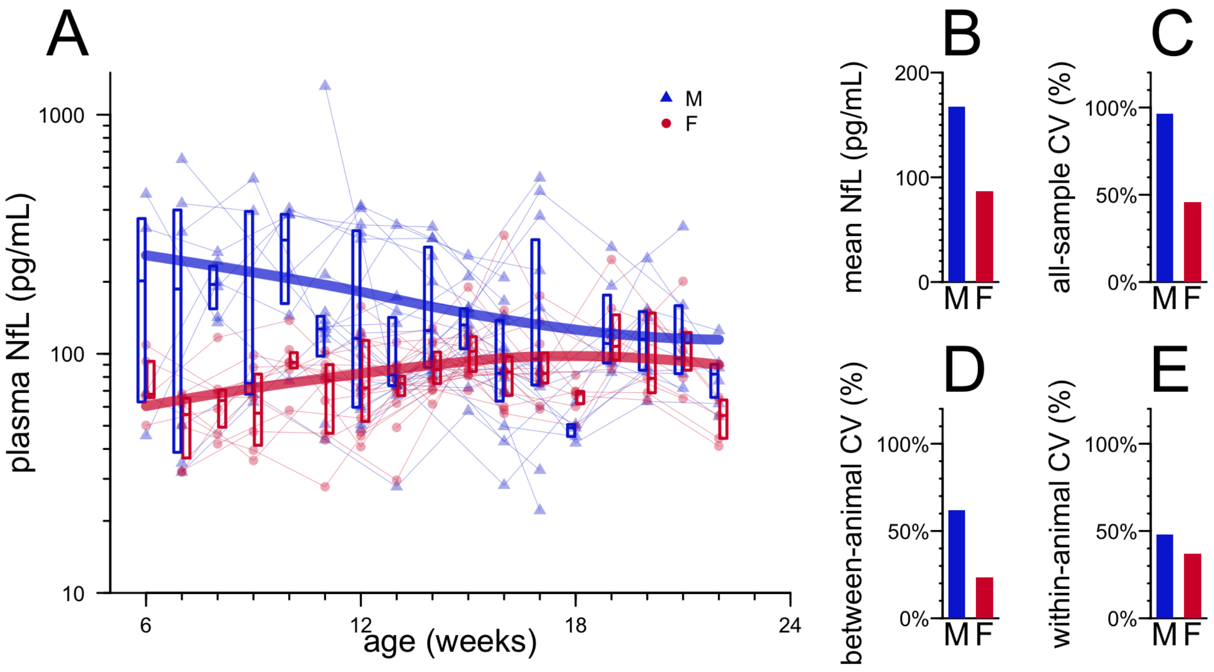

Supplementary Figure 2. Natural history of plasma NfL in naive C57BL/6N mice. A) *Plasma NfL values from N = 272 serial bleeds. Points represent individual animals (blue triangles for males, red circles for females), thin lines connect serial samples from individual animals, boxes represent medians and interquartile ranges by sex and timepoint, and thick bars represent locally estimated scatterplot smoothing (LOESS) fits by sex.* ***B)*** *Mean NfL values by sex across all animals, all timepoints. N=24 per sex.* ***C)*** *Coefficient of variation by sex across all samples, all timepoints. N=24 per sex.* ***D)*** *Mean coefficient of variation by sex, between animals within each timepoint. N=24 per sex.* ***E)*** *Mean coefficient of variation by sex, within animal between timepoints. N=24 per sex.*

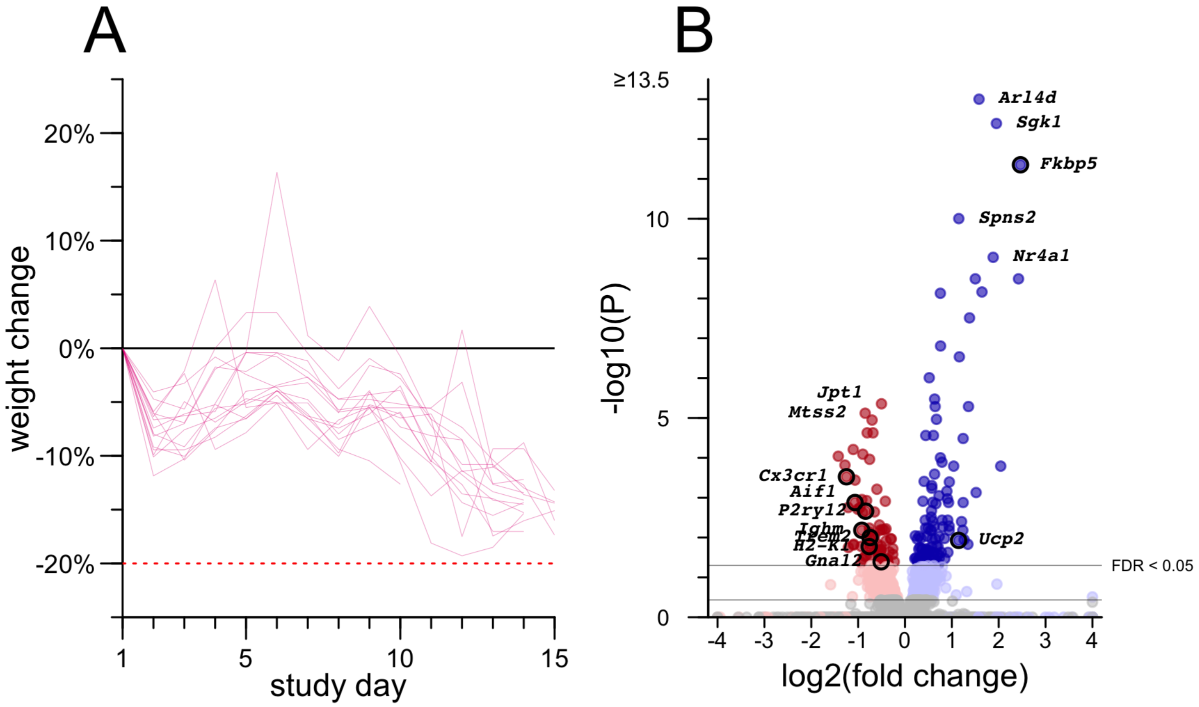

Supplementary Figure 3. Additional results from mouse study 1. *Left: individual weight trajectories for N=16 mice treated with 50 mg/kg/day minocycline, normalized to individual baseline. Each line is one mouse. Right: volcano plot of differentially expressed genes in the brains of these mice (N=3 treated and N=8 untreated) by bulk RNA-seq analyzed with DESeq2, see Methods for details. Each point is one gene symbol.*

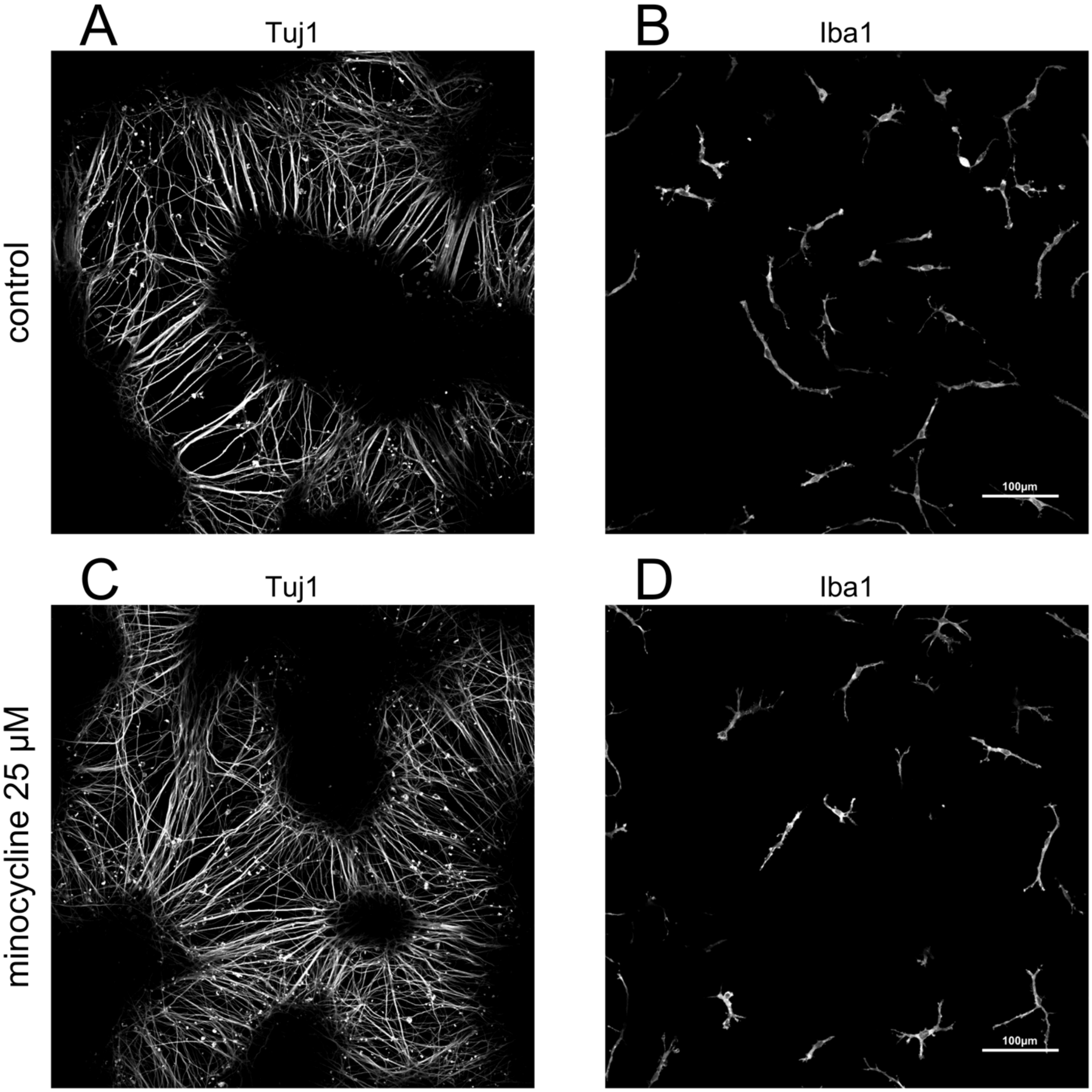

Supplementary Figure 4. Co-culture images broken out by color channel. *Control (A-B) and minocycline (C-D) images from Figure 5, broken out by green Tuj1 channel (A, C) and magenta Iba1 channel (B, D).*

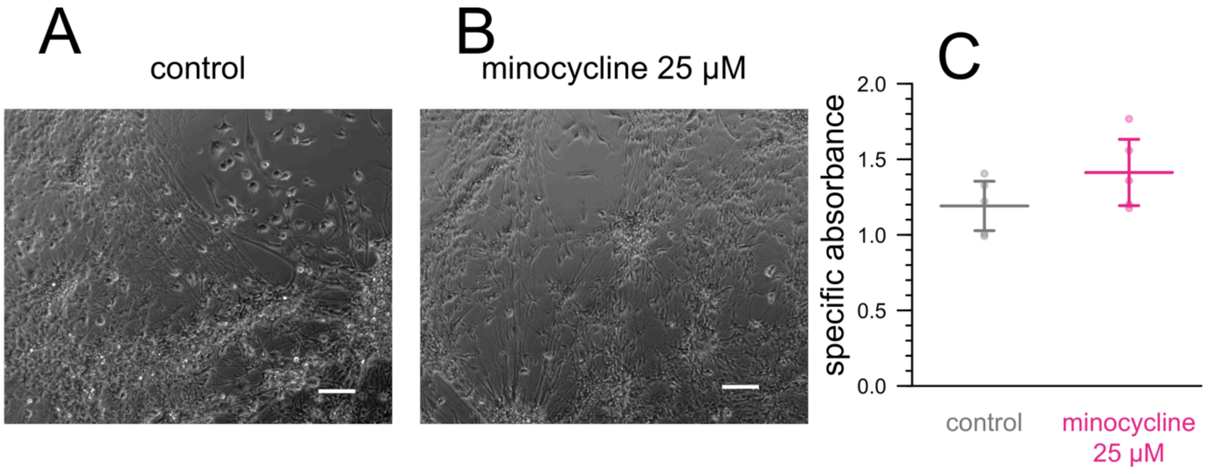

Supplementary Figure 5. Viability assessment of minocycline-treated and control co-cultures. *5 new wells of cells per condition were grown, treated with 25 μM minocycline or control for 6 days, then brightfield images were taken (A-B). Media was collected and assessed for specific absorbance (C; see Methods) to determine cell viability. No difference between control and minocycline-treated cultures was noted. Each point represents one well, N=5 per group. (P = 0.15, 2-sided T test). Scale bar, 100 μm.*

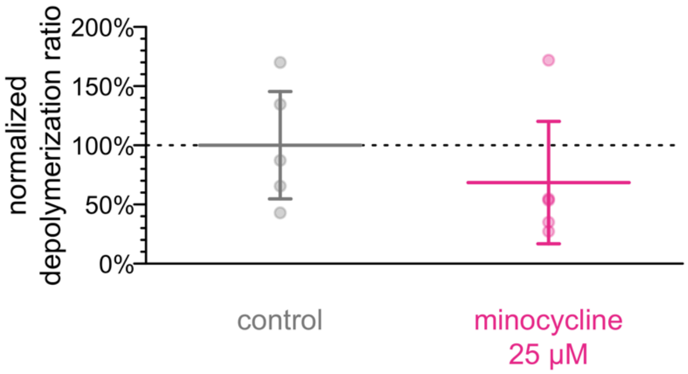

Supplementary Figure 6. Neurite disintegrity quantification for minocycline-treated and control co-cultures. *Cells were imaged as described in Methods > Immunocytochemistry and microscopy the normalized axonal depolymerization ratio was calculated for each well. N=5 wells of co-cultures per treatment condition (N=3 from cells imaged in Figure 4 and N=2 images from second replication experiment conducted specifically to quantify this ratio). No difference*

between control and minocycline-treated cultures was noted ($P = 0.31$, 2-sided Wilcoxon test).

Supplementary References

1. Moore RA, Ward A, Race B, Priola SA. Processing of high-titer prions for mass spectrometry inactivates prion infectivity. *Biochim Biophys Acta Proteins Proteom*. 2018;1866(11):1174-1180. doi:10.1016/j.bbapap.2018.08.004

2. Minikel EV, Kuhn E, Cocco AR, et al. Domain-specific quantification of prion protein in cerebrospinal fluid by targeted mass spectrometry. *Mol Cell Proteomics*. Published online September 26, 2019. doi:10.1074/mcp.RA119.001702

3. Mortberg MA, Zhao HT, Reidenbach AG, et al. Regional variability and genotypic and pharmacodynamic effects on PrP concentration in the CNS. *JCI Insight*. 2022;7(6):e156532. doi:10.1172/jci.insight.156532

4. Hughes CS, Foehr S, Garfield DA, Furlong EE, Steinmetz LM, Krijgsveld J. Ultrasensitive proteome analysis using paramagnetic bead technology. *Mol Syst Biol*. 2014;10(10):757. doi:10.15252/msb.20145625

5. Hughes CS, Moggridge S, Müller T, Sorensen PH, Morin GB, Krijgsveld J. Single-pot, solid-phase-enhanced sample preparation for proteomics experiments. *Nat Protoc*. 2019;14(1):68-85. doi:10.1038/s41596-018-0082-x

6. Erickson BK, Mintseris J, Schweppe DK, et al. Active Instrument Engagement Combined with a Real-Time Database Search for Improved Performance of Sample Multiplexing Workflows. *J Proteome Res*. 2019;18(3):1299-1306. doi:10.1021/acs.jproteome.8b00899

7. Eng JK, McCormack AL, Yates JR. An approach to correlate tandem mass spectral data of peptides with amino acid sequences in a protein database. *J Am Soc Mass Spectrom*. 1994;5(11):976-989. doi:10.1016/1044-0305(94)80016-2

8. Huttlin EL, Jedrychowski MP, Elias JE, et al. A tissue-specific atlas of mouse protein phosphorylation and expression. *Cell*. 2010;143(7):1174-1189. doi:10.1016/j.cell.2010.12.001

9. Elias JE, Gygi SP. Target-decoy search strategy for increased confidence in large-scale protein identifications by mass spectrometry. *Nat Methods*. 2007;4(3):207-214. doi:10.1038/nmeth1019
